# Glyphosate-based herbicides at low doses affect canonical pathways in estrogen positive and negative breast cancer cell lines

**DOI:** 10.1371/journal.pone.0219610

**Published:** 2019-07-11

**Authors:** Elaine Stur, Andrés Felipe Aristizabal-Pachon, Kamila Chagas Peronni, Lidiane Pignaton Agostini, Sabine Waigel, Julia Chariker, Donald M. Miller, Shelia Dian Thomas, Francine Rezzoug, Raquel Spinassé Detogni, Raquel Silva dos Reis, Wilson Araujo Silva Junior, Iuri Drumond Louro

**Affiliations:** 1 Programa de Pós-graduação em Biotecnologia, Universidade Federal do Espírito Santo, Vitória, Espírito Santo, Brasil; 2 Departamento de Ciências Biológicas-Núcleo de Genética Humana e Molecular, Universidade Federal do Espírito Santo, Vitória, Espírito Santo, Brasil; 3 Department of Genetics at Ribeirão Preto Medical School, and Center for Medical Genomics - HCRP, University of São Paulo, Ribeirão Preto, São Paulo, Brazil; 4 National Institute of Science and Technology in Stem Cell and Cell Therapy and Center for Cell-Based Therapy, Ribeirão Preto, São Paulo, Brazil; 5 Molecular Targets Program, JG Brown Cancer Center, University of Louisville, Louisville, Kentucky; 6 Department of Computer Engineering and Computer Science, Speed School of Engineering, University of Louisville, Kentucky, United States of America; 7 James Graham Brown Cancer Center, Department of Medicine, University of Louisville, Louisville, Kentucky, United States of America; University of South Alabama Mitchell Cancer Institute, UNITED STATES

## Abstract

Glyphosate is a broad-spectrum herbicide that is used worldwide. It represents a potential harm to surface water, and when commercially mixed with surfactants, its uptake is greatly magnified. The most well-known glyphosate-based product is Roundup. This herbicide is potentially an endocrine disruptor and many studies have shown the cytotoxicity potential of glyphosate-based herbicides. In breast cancer (BC) cell lines it has been demonstrated that glyphosate can induce cellular proliferation via estrogen receptors. Therefore, we aimed to identify gene expression changes in ER+ and ER- BC cell lines treated with Roundup and AMPA, to address changes in canonical pathways that would be related or not with the ER pathway, which we believe could interfere with cell proliferation. Using the Human Transcriptome Arrays 2.0, we identified gene expression changes in MCF-7 and MDA-MB-468 exposed to low concentrations and short exposure time to Roundup Original and AMPA. The results showed that at low concentration (0.05% Roundup) and short exposure (48h), both cell lines suffered deregulation of 11 canonical pathways, the most important being cell cycle and DNA damage repair pathways. Enrichment analysis showed similar results, except that MDA-MB-468 altered mainly metabolic processes. In contrast, 48h 10mM AMPA showed fewer differentially expressed genes, but also mainly related with metabolic processes. Our findings suggest that Roundup affects survival due to cell cycle deregulation and metabolism changes that may alter mitochondrial oxygen consumption, increase ROS levels, induce hypoxia, damage DNA repair, cause mutation accumulation and ultimately cell death. To our knowledge, this is the first study to analyze the effects of Roundup and AMPA on gene expression in triple negative BC cells. Therefore, we conclude that both compounds can cause cellular damage at low doses in a relatively short period of time in these two models, mainly affecting cell cycle and DNA repair.

## Introduction

Glyphosate (*N*-(phosphonomethyl) glycine) is a broad-spectrum herbicide used worldwide in agriculture and forestry for weed control, representing a potential harm to surface water, ground waters and sediments [1–6]. It has been commercialized since 1974, as Glyphosate-Based Herbicides (GBHs). The commercialized products are often formulated with other materials, such as surfactants, with the most well-known brand name being Roundup (Monsanto Co, St Louis, MO, USA) [7].

Roundup is a powerful herbicide due to its action in the shikimate pathway targeting the enzyme 5-enolpyruvyl-3-shikimate phosphate synthase (EPSPS), a production pathway of essential aminoacids, such as tryptophan, phenylalanine and tyrosine [8,9]. The shikimate pathway only exists in plants, bacteria and some fungi, which may explain the reported low toxicity in mammals (LD_50_ >4g/kg in humans). Because of these characteristics, glyphosate has been the most successful herbicide in history [4,5].

Adjuvants used to produce Roundup are surfactants that facilitate glyphosate uptake in cells through plasma membranes, increasing efficiency, stability and bioaccumulation [10]. The exact formulation of surfactants is not available, but the most common is polyethoxylated tallow amine (POEA) or alkylamine [11]. Many studies demonstrate that Roundup is more efficient than its active principle, suggesting a synergistic effect caused by adjuvants [1,2,10].

Glyphosate is degraded in the soil by microorganisms to aminomethylphosphonic acid (AMPA) [12,13]. In mammals glyphosate is not completely degraded and is eliminated unchanged in urine, but some studies have shown that glyphosate can be degraded by gut bacteria [13,14]. There is no consensus about AMPA action in mammals, but it is known that after glyphosate is metabolized, AMPA accounts for only 0.2–0.3% of the initial volume. While glyphosate is the most commercialized herbicide in the world, AMPA has no commercial use [12].

Although currently considered by the International Agency for Research on Cancer (IARC) as a class 2A carcinogen (probably carcinogenic), there is no consensus about how toxic GBHs are to mammals. Typically, GBHs are used in concentrations between 1–2%, or 21-42mM of glyphosate, but in some cases, even lower concentrations can have an impact on human health [4,10]. Nevertheless, there is limited evidence that glyphosate is carcinogenic. Human *in vitro* studies and animal models have shown genotoxic potential, chromosomal damage, and oxidative stress induction [13]. In addition, GBH may disrupt estrogen synthesis [10] through aromatase deregulation. Therefore, glyphosate may affect diseases related to hormone physiology, such as breast cancer (BC) [1].

Thongprakaisang (2013) and Mesnage et al (2017) showed glyphosate stimulation of estrogen receptor (ER) in ER+ BC cell lines, but not in ER-, in a dose-dependent manner. Hokanson et al (2005) showed a similar effect of glyphosate and estrogen in ER+ cells. De Almeida et al (2018) showed that low concentrations of glyphosate alone and Roundup did not show significant effects on viability in MDA-MB-231 and MCF-7 cells, but observed an increase in DNA damage with the Roundup formulation [15]. The effect of GBHs on ER- BC cells is not well understood. Therefore, we aimed to identify gene expression changes in ER+ and ER- BC cell lines treated with low concentration and short time of Roundup and AMPA, to address changes in canonical pathways, which we believe could interfere with cell proliferation.

## Materials and methods

### Chemicals

(Aminomethyl) phosphonic acid (AMPA, CAS Number: 1066-51-9), 3-(4,5-dimethylthiazol-2-yl)-2,5-diphenyl tetrazolium bromide (MTT) and dimethyl sulfoxide (DMSO) were purchased from Sigma Aldrich (St. Louis, Missouri, EUA). The Roundup Original herbicide formulation (N—(Phosphonomethyl) glycine Isopropylamine salt—480 g/L and 360g/L of glyphosate) (Monsanto, São Paulo, Brazil) was available on the market.

### Cell lines and culture conditions

A hormone-independent human breast cancer cell line MDA-MB-468 (ER-) and a hormone-dependent human BC cell line MCF-7 (ER+), were obtained from the American Type Collection (ATCC), USA. The MDA-MB-468 were cultured in RPMI 1640 (1X) and MCF-7 were cultured in DMEM (1X), both supplemented with 10% Fetal Bovine Serum (FBS) and 1% Penicillin-streptomycin. Cells were maintained at 37°C in humidified environment with 95% air and 5% CO_2_. Culture medium and supplements were purchased from Gibco-Invitrogen Life Technology (Carlsbad, CA, USA).

### Cell viability MTT Assay

Cell growth and viability were tested using the MTT reagent assay. Cells were seeded at 5X10^3^cells /100μL/well in 96-well microtiter plates. After 24h incubation for attachment, cells were treated with concentrations of AMPA ranging from 0.01 to 10mM and 0.01% to 0.3% of Roundup. After 3h, 15h, 24 and 48h of incubation period, the medium was removed and 0.5mg/mL of MTT was added into each well. Cells were incubated for 3h, then the medium was removed and 100μL of DMSO was added to each well to dissolve the precipitated dye. After 1 hour, the changes were measured by optical density at 570 nm, using microplate readers FLUOstar Omega (BGM LabTech). Cell sensitivity to a chemical was expressed as the % cell viability compared to control cells. All the experiments were made in biological and technical triplicates.

### RNA extraction

Total RNA was extracted from biological samples treated with Roundup at 0.05% (1.1mM of glyphosate) and AMPA at 10mM for 48 hours, using RNeasy Mini Kit (Qiagen, Hilden, Germany) according to the manufacturer’s recommendations. RNA purity was assessed using a NanoDrop 2000 (ThermoFisher Scientific, Waltham, Massachusetts, EUA) and RNA quality was checked using Agilent Bioanalyzer 2100 (Agilent Technologies, Santa Clara, CA). Only RNA preparations with an RNA integrity number (RIN) >7 were considered for microarray analysis. All samples were analyzed in triplicates.

### Transcriptome profiling

RNA was analyzed using the Human Transcriptome Arrays 2.0 (HTA 2.0), which evaluates more than 67,500 coding and non-coding transcripts, with more than six million oligonucleotide probes (25 bases in length). This array is able to interrogate all transcript isoforms in the human transcriptome with probes targeting coding transcripts, exon-exon splice junctions and non-coding transcripts [16,17].

All procedures were performed according to the manufacturer’s protocols. From each sample, complementary RNA (cRNA) was prepared from 100ng of total RNA according to the Affymetrix Whole Transcript (WT) protocol. cRNA was used to generate single-stranded DNA, which was fragmented and biotinylated. The labeled single-stranded DNA was hybridized for 16 h at 45 °C on the Affymetrix HTA 2.0 microarrays. Then, microarrays were washed and stained with a streptavidin-phycoerythrin conjugate in the Affymetrix Fluidics Station 450. Scanning and data extraction were followed by the transformation of fluorescence data into CEL files employing the Affymetrix GeneChip Command Console (AGCC) software [16,18].

### Data analysis

Data were analyzed using the Partek Genomics Suite v 6.6 (Partek Inc., Louis, MO). Principal Component Analysis (PCA) was applied to assess sample distribution. Pre-processing of Affymetrix CEL-files was performed using the robust multi-chip analysis (RMA) algorithm, which performs background adjustment, quantile normalization and probe summarization. Differential expression analysis was realized using a one-way ANOVA. Class comparison was performed using ANOVAs, which included a multiple testing correction using False Discovery Rates (FDRs) set with a p-value <0.01 considered as significant for biological and molecular function analyses. Up- and down-regulated genes were identified using a fold-change of ≥1.5 and ≤ −1.5.

Enrichment analysis (EA) was performed using MetaCore (GeneGo, Thomson Reuters, NY), where genes with altered expression were mapped to Gene Ontology (GO). GO annotations were used as indicators of biological functions. GO describes gene products in terms of their associated biological processes, cellular components, and molecular functions [19]. Pathview (Luo et al. 2013) was used to construct canonical pathway maps.

## Results

### Cell viability evaluation

**Fig 1:**
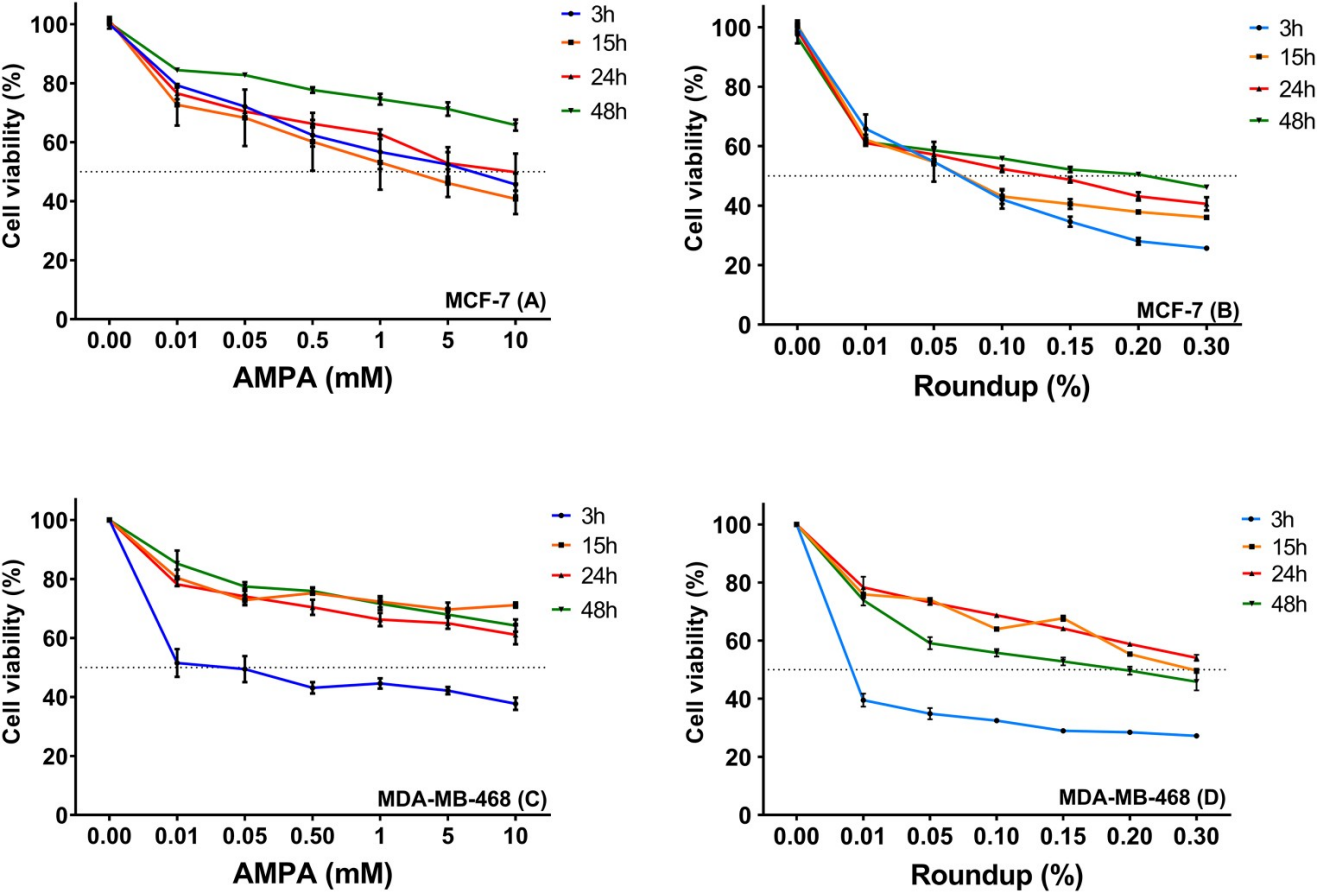
Results of cell viability (MTT assay) of MCF-7 and MDA-MB-468 after treatments with Roundup and AMPA. Controls for both groups were untreated cells. AMPA concentrations were between 0.01-10mM. Roundup dilution was based on % (v/v), and concentrations of 0.01%-0.30% were used.

The cell viability assay (MTT) is shown in Fig 1. It is possible to observe that both cell lines exhibited similar patterns under Roundup and AMPA treatments. In MCF-7 cells, 0.15% Roundup caused more cell death at 3 hours than at 48 hours, and at 0.3% concentration less than 30% of the cells survived. Similar responses were found for MDA-MB-468, in which the 3 hour-treatment was more toxic than all other time points for all concentrations. At 48 hours using the highest concentration (0.3% Roundup), survival was less than 50% for both cell lines.

AMPA effects at 48h were less toxic than at 3h, for all concentrations on both cell lines. Similar results were observed for 10mM/48 hours, with a survival rate of approximately 70%. In MCF-7, a 15 hour-treatment cause more death, and in MDA-MB-468 the 3-hour exposure caused the most death, for all concentrations. Observed cell death levels at 3 hours could be an effect of the type of assay used (MTT) that measures the mitochondrial metabolic rate and indirectly reflects viable cell numbers.

### Differential gene expression

**Fig 2:**
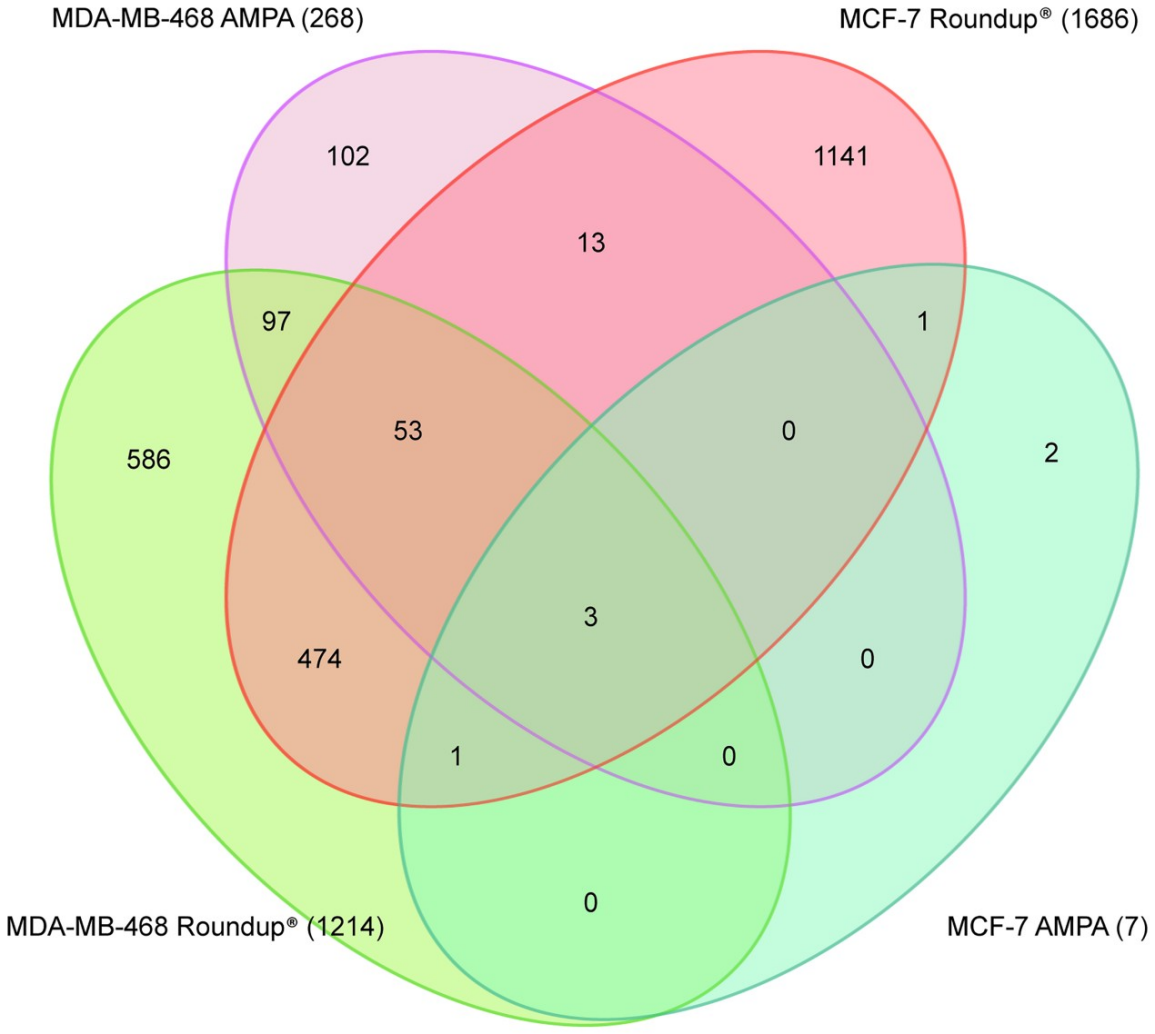
Venn diagram. The figure shows the number of common DEGS for both cell lines and treatments. Only 3 genes were common among all conditions.

**Fig 3:**
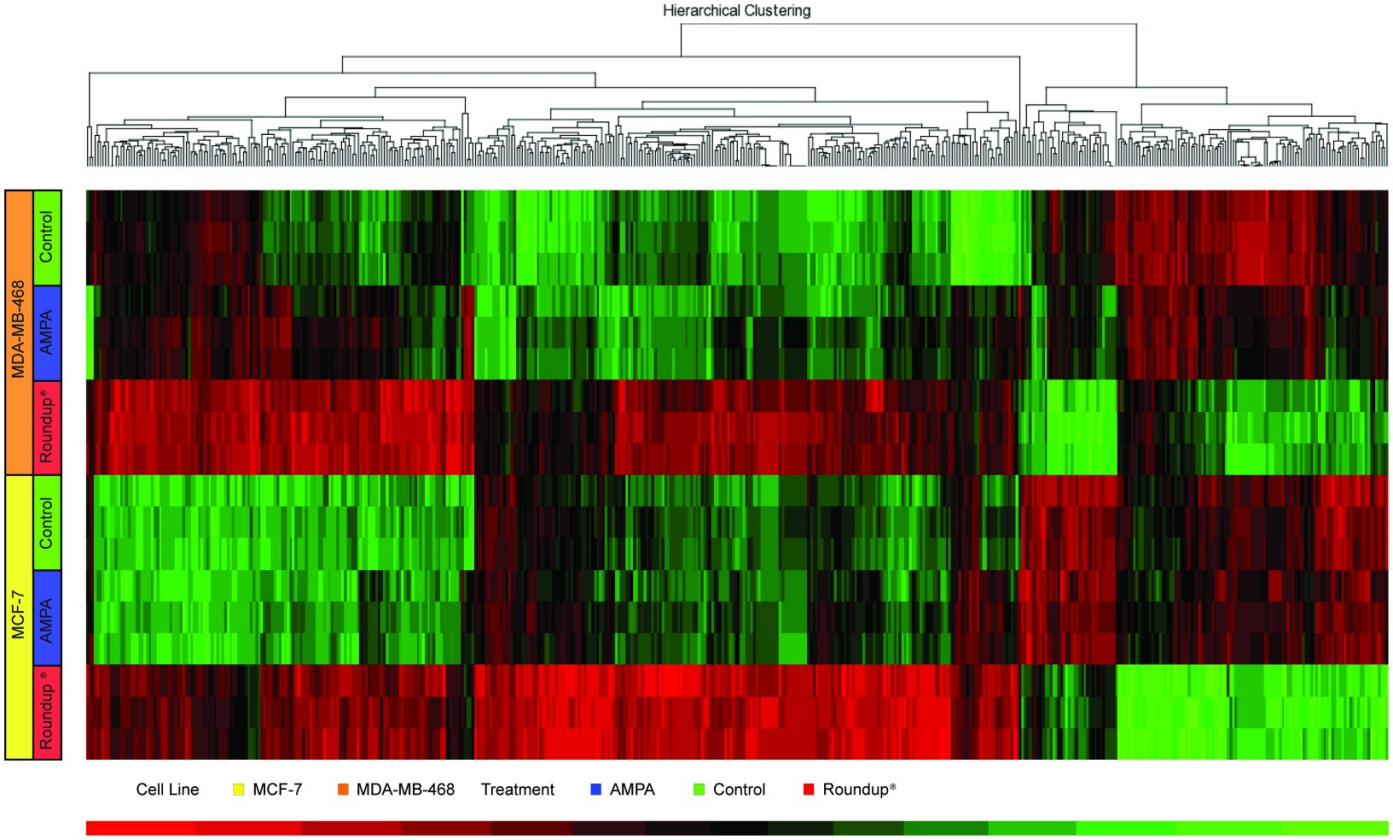
The hierarchical distribution of the Top 500 DEGs.

A 48-hour treatment of 1.1mM glyphosate (0.05%) and 10mM AMPA was chosen to analyze gene expression. Differentially expressed genes (DEGs) were identified using a p-value adjusted (FDR) of <0.01 and a minimal fold change of 1.5. There were 1,686 DEGs identified in MCF-7 after Roundup exposure and 7 DEGs after AMPA treatment. There were 1,214 DEGs identified in Roundup-treated MDA-MB-468 and 268 DEGs after AMPA treatment. In Fig 2, there were 531 common DEGs for Roundup and 3 for AMPA, which were downregulated for all treatments and cell lines (*BNIP3*, *FAM162A* and *PGK1*) (Table 1). Fig 2, a Venn diagram for all treatments and cell lines, and Fig 3, a hierarchical distribution of the top 500 DEGs, show Roundup exposure leading to different gene expression patterns when compared to AMPA exposure and control.

**Table 1:** Differentially expressed genes, downregulated in two cell lines after Roundup and AMPA treatments.

	Fold-change			
Gene Symbol	MDA- AMPA	MDA- Roundup	MCF-7- AMPA	MCF-7- Roundup
*BNIP3*	-3.39378	-3.19528	-2.08295	-1.94162
*FAM162A*	-1.97305	-2.36339	-2.02376	-2.48821
*PGK1*	-2.30474	-4.52354	-1.71906	-3.93362

### Enrichment analysis

Comprehensive process networks analysis of deregulated genes after Roundup treatment revealed their association with cell cycle and DNA damage repair. MCF-7 GO (Gene Ontology) cellular process analysis showed changes related to cell cycle and cell metabolism processes, whereas MDA-MB-468 cell changes were related to metabolism. Table 2 displays the top 10 processes networks and GO cellular processes.

**Table 2:** Top 10 process networks and GO cellular processes to MCF-7 and MDA-MB-468 treated with Roundup.

**MCF-7- Roundup**			
***Process Network***	***FDR***	***GO Process***	***FDR***
Cell cycle_S phase	1.90E-18	Cell cycle	3.943E-41
DNA damage_Checkpoint	5.411E-14	Cellular metabolic process	1.067E-40
Cell cycle_Core	2.114E-11	Mitotic cell cycle	2.625E-40
Cell cycle_G1-S	4.673E-07	Cell cycle process	9.226E-38
Cell cycle_G2-M	5.459E-07	Cellular macromolecule metabolic process	1.555E-36
DNA damage_DBS repair	3.781E-06	Cellular response to stress	1.652E-36
Cell cycle_Mitosis	9.690E-06	Nucleobase-containing compound metabolic process	1.100E-35
DNA damage_MMR repair	4.794E-05	Organic cyclic compound metabolic process	3.301E-35
Apoptosis_Apoptotic nucleus	4.794E-05	Mitotic cell cycle process	7.841E-35
Cell cycle_Meiosis	2.837E-04	Nucleic acid metabolic process	1.242E-34
**MDA-MB-468- Roundup**			
***Process Network***	***FDR***	***GO Process***	***FDR***
Cell cycle_S phase	5.484E-05	Cellular metabolic process	1.153E-16
DNA damage_Checkpoint	4.621E-03	Primary metabolic process	1.170E-15
Reproduction_FSH-beta signaling pathway	1.915E-02	Cellular macromolecule metabolic process	3.385E-15
Cell cycle_G1-S	1.915E-02	Metabolic process	3.178E-14
Cell cycle_G2-M	1.915E-02	Organic substance metabolic process	1.328E-13
Apoptosis_Apoptotic nucleus	2.516E-02	Cellular process	3.276E-13
Protein folding_Response to unfolded proteins	2.516E-02	Nitrogen compound metabolic process	7.355E-13
Cytoskeleton_Intermediate filaments	2.516E-02	Mitotic cell cycle	7.355E-13
Development_Blood vessel morphogenesis	3.680E-02	Regulation of cellular metabolic process	6.546E-12
Proliferation_Negative regulation of cell proliferation	3.680E-02	Cell cycle	1.088E-11

There was no significant pathway enrichment for either cell line exposed to AMPA. However, some enrichment was found for GO cellular processes (see Table 3). MDA-MB-468 was enriched for doxorubicin metabolic process and MCF-7 was enriched for response to abiotic stimulus.

**Table 3:** Statistically significant GO cellular processes for MCF-7 and MDA-MB-468 treated with AMPA.

**MCF-7- AMPA**	
***GO Process***	***FDR***
Response to abiotic stimulus	3.701E-03
NADH regeneration	3.701E-03
Glucose catabolic process to pyruvate	3.701E-03
Canonical glycolysis	3.701E-03
Glycolytic process through glucose-6-phosphate	3.701E-03
Glycolytic process through fructose-6-phosphate	3.701E-03
Glucose catabolic process	3.701E-03
Positive regulation of release of cytochrome c from mitochondria	3.701E-03
Cellular response to hypoxia	3.701E-03
Cellular response to decreased oxygen levels	3.826E-03
**MDA-MB-468- AMPA**	
***GO Process***	***FDR***
Doxorubicin metabolic process	7.606E-08
Daunorubicin metabolic process	7.606E-08
Polyketide metabolic process	7.606E-08
Aminoglycoside antibiotic metabolic process	1.246E-07
Single-organism cellular process	1.859E-07
Small molecule biosynthetic process	1.859E-07
Cellular response to decreased oxygen levels	4.617E-07
Cellular response to oxygen levels	9.563E-07
Alcohol biosynthetic process	9.844E-07
Single-organism biosynthetic process	1.072E-06

When looking at MCF-7 unique differentially expressed genes, cellular component organization or biogenesis is the main altered GO cellular process, which is responsible for biosynthesis of constituent macromolecules, assembly, and arrangement of constituent parts or disassembly of cellular components. The second GO cellular process enriched for this cell line was cell cycle. Table 4 lists the top significantly enriched GO cellular processes for each cell line. In the process network analysis, the main process was cell cycle.

**Table 4:** Results of enrichment analysis (GO cellular processes) after exclusion of common genes from Roundup.

MCF-7- Roundup		MDA-MB-468- Roundup	
*GO Process*	*FDR*	*GO Process*	*FDR*
Cellular component organization or biogenesis	5.555E-29	Regulation of cellular metabolic process	1.415E-13
Cell cycle	5.977E-28	Tissue development	1.415E-13
Organic cyclic compound metabolic process	2.161E-26	Regulation of macromolecule metabolic process	3.462E-13
Cellular component organization	6.472E-26	Regulation of nitrogen compound metabolic process	7.725E-13
Cellular metabolic process	1.651E-25	Anatomical structure morphogenesis	7.725E-13
Nucleobase-containing compound metabolic process	4.998E-25	Regulation of metabolic process	7.725E-13
Cellular nitrogen compound metabolic process	1.727E-24	Tissue morphogenesis	1.360E-12
Nucleic acid metabolic process	1.764E-24	Regulation of primary metabolic process	2.495E-12
Cellular aromatic compound metabolic process	6.957E-24	Single-organism cellular process	2.495E-12
Heterocycle metabolic process	8.398E-24	Anatomical structure formation involved in morphogenesis	3.331E-12

The top 10 GO cellular processes of MDA-MB-468 unique differentially expressed genes did not include cell cycle. However, most processes were related to metabolism, which was confirmed by the process networks analysis (Table 4).

Despite the low number of common DEGs (3 genes), the EA indicates that GO cellular process response to hypoxia was significantly enriched. A Metacore pathway enrichment analysis indicates a significant enrichment in the transcription of *HIF-1* targets pathway (see Fig A in S1 File), which included 2 out of the 3 DEGs common to both cell lines (*BNIP3* and *PGK1*).

### Canonical pathways

**Fig 4:**
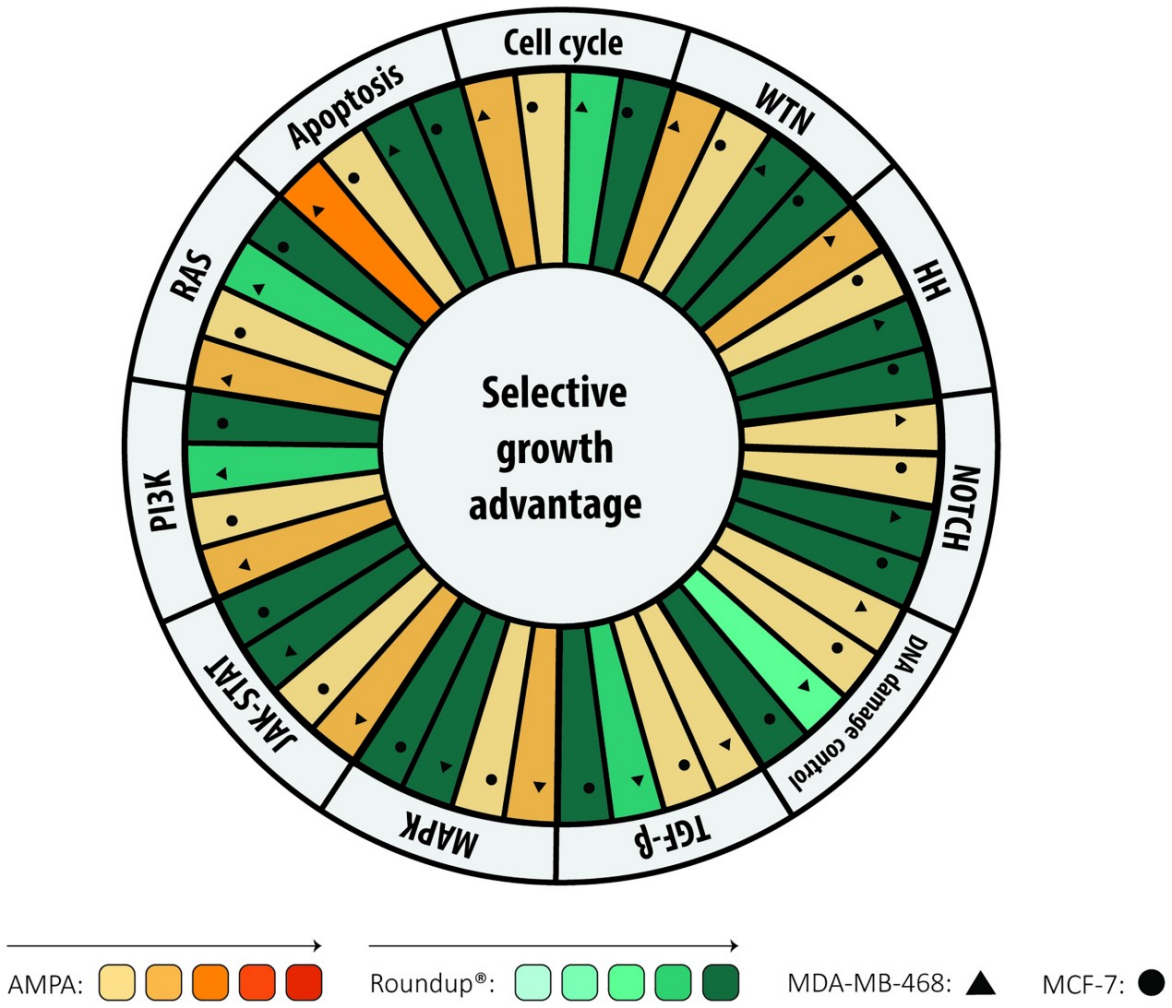
Figure shows 11 canonical pathways included in the DEGS found for both cell lines and treatments. Color intensity correlates with pathway alteration levels.

Using Pathview web, the effect of DEGs in 11 canonical pathways was analyzed: NOTCH, WNT, Hedgehog, TGF-β, MAPK, JAK-STAT, PI3K-AKT, RAS, Cell cycle, Apoptosis and DNA damage control. Roundup treatment showed deregulation of all these pathways in both cell lines. AMPA-treated MDA-MB-468 showed some DEGs in WNT, Hedgehog, MAPK, JAK-STAT, PI3K, RAS, Cell cycle and Apoptosis, which were not found in MCF-7. Fig 4 shows pathway changes in the two cell lines and treatments.

As previously reported in EA, cell cycle was the most affected process. MDA-MB-468 exhibited changes when exposed to both treatments, while MCF-7 exhibited changes only with Roundup (see Fig B in S1 File). In MCF-7, pathway deregulation is associated with downregulation of key genes, such as *c-MYC*, cyclins and *PCNA*. MDA-MB-468 was also deregulated, however, fewer genes were affected.

Several pathways related to DNA damage repair, base excision repair, nucleotide excision repair and mismatch repair, were included in the analysis. None of them were altered by AMPA treatment. In comparison, Roundup treatment affected all of them, especially base excision repair, where affected genes were all downregulated. DNA damage repair pathways and the complete deregulated gene list for 11 canonical pathways are shown in the supplementary materials (Fig C-E and Tables A-M in S1 File).

## Discussion

As previously described in the literature, glyphosate can potentially induce cell proliferation of ER+ BC cell lines, whereas Roundup (the complexed herbicide), leads to cell death, due to a toxic cellular effect. Our aim was to show gene expression changes in ER+ BC cell line (MCF-7) and in a triple negative BC cell line (MDA-MB-468) exposed to Roundup and AMPA to understand if the effect of these chemicals are dependent on cell ER positivity.

Concentrations used here were lower than the manufacturer’s concentration recommendation for agricultural use. Roundup Original is diluted in water in concentrations between 0.5%-2% depending on the specific product. In Brazil, the ANVISA, (National Healthy Surveillance Agency), allows the maximum use of 1% (1.0ppm). The US FDA currently allows for the use of 0.1 to 310ppm [20,21]. We intended to use lower concentrations than allowed by controlling agencies. In our results it is possible to observe that low concentrations of Roundup Original showed high levels of cell death on both cell lines at all time-points. These results are similar to the findings of Mesnage et al. (2017), where Roundup did not cause a significant increase in cell proliferation and had a more toxic effect than glyphosate [22]. Other studies have demonstrated the same effect in other cell lines. Benachour et al. (2007) showed that Roundup formulations are more toxic to HEK293 (human embryonic kidney) than glyphosate alone [10]. Moreover, Richard et al. (2005) analyzed the JEG3 cell line (choriocarcinoma), showing high toxicity after Roundup treatment [2].

Our data show that AMPA is less toxic than Roundup. Similar results were found by Li et al. (2013) where 8 human cancer and 2 immortalized human cell lines treated with AMPA at 50mM were analyzed. Their results showed inhibition of cell proliferation of all cancer cell lines, but not non-immortalized ones, suggesting that highly proliferative cancer cells are more sensitive to these drugs than slower ones [12].

AMPA is a glycine analog which may decrease intracellular glycine synthesis through inhibition of serine hydroxymethyltransferase (SHMT), the enzyme responsible to catalyze the conversion of glycine to serine and vice-versa. This occurs because SHMT catalyzes the production of one-carbon units used for *de novo* thymidylate biosynthesis [12,23,24]. Rapidly proliferating cancer cells show a high demand of glycine, one third of which comes from extracellular sources, whereas slowly proliferating cancer cells are self-sufficient for glycine production [12,25]. Therefore, a reduction in glycine production may have a toxic effect on rapidly proliferating tumor cells, which could explain viability results found here.

After Roundup treatment, analysis of 11 canonical pathways and association with EA showed that cell cycle and DNA damage repair are affected, and that this compound is more toxic to MCF-7. Chaufan et al. (2014) analyzed the effects of glyphosate, GBHs and AMPA on HepG2 (liver hepatocellular cells), finding that AMPA and glyphosate alone did not affect cell viability in a concentration up to 1000mg/L, while GBHs caused rapid cell death. In addition, GBHs induced cytotoxicity, reactive oxygen species (ROS) production and apoptosis at low doses [26]. In a similar fashion, Mesnage et al. (2017) analyzing glyphosate treated MCF-7 cell gene expression found cell cycle alterations [22], whereas Hokanson et al. (2005) reported changes in the HIF-1 pathway [4].

De Almeida et al. (2018) also explored the effects of GBH and glyphosate in ER+ and ER- BC cell lines. Their results showed that these compounds can cause DNA damage at low concentrations and short exposure [15], as proposed in our study. In addition, they concluded that glyphosate and formulations may be able to induce cell damage, independently of estrogenic pathways, corroborating our findings.

Studies with sea urchin have shown that Roundup is able to cause a delay in cell cycle progression after 30 minutes of treatment, suggesting that the delay in activation of CDK1/cyclin B complex is responsible for the deregulation of cell cycle progression [27]. Similarly, in 2004, Marc et al. showed that in Roundup treated sea urchin embryos, DNA synthesis was inhibited by 75%, possibly due to the same mechanism [28].

Our results show downregulation of cyclins and DNA damage repair pathways. Based on pathway analysis, main cell cycle changes could happen in G1 and S phases (Fig B in S1 File). Our findings suggest that Roundup affects survival due to changes in cell cycle regulation and metabolism, which could alter mitochondria oxygen consumption, increase ROS levels, induce hypoxia, disrupt DNA repair, cause mutation accumulation, and ultimately lead to cell death. Roundup’s higher toxicity is probably due to the addition of surfactants in its formula, which may explain the lack of cell proliferation after treatment with it [22].

To our knowledge, this is the first study that analyzes the effects of Roundup and AMPA on gene expression in triple negative BC cells. EA analysis shows that independently of ER positivity, the two cell lines can suffer deregulation of cell cycle and DNA repair.

MDA-MB-468 changes were more related to metabolism, whereas MCF-7 changes were more linked to cell cycle. Considering both cell lines doubling time (MCF-7: 24 hours, MDA-MB-468: 48 hours), it is possible that our 48-hour treatment allowed MCF-7 cells to divide twice and show changes in the cell cycle more clearly than MDA-MB-468 cells.

In summary, we can conclude that Roundup, at much lower doses than the ones used in agriculture, was able to deregulate important intracellular pathways in ER+ and triple negative BC cell lines, showing that glyphosate’s effect on cells is not exclusive to the ER pathway. Alterations in gene expression of cell cycle pathways were more prominent on MCF-7 than MDA-MB-468 cells. In contrast, AMPA exposure had a higher effect on MDA-MB-468 than MCF-7 cells.

## Supporting information

S1 File (DOC)
