# Supplementary material for: Glyphosate-based herbicides at low doses affect canonical pathways in estrogen positive and negative breast cancer cell lines
Source: PLoS One. 2019 Jul 11;14(7):e0219610. doi: 10.1371/journal.pone.0219610 (PMC6622539; doi:10.1371/journal.pone.0219610)
Supplement: S1 File — (DOC) [file pone.0219610.s001.doc]

Table A: Notch signaling altered by AMPA and Roundup treatments in MDA-MB-468 and MCF-7.

| **04330-Notch signaling pathway** | | | |
| --- | --- | --- | --- |
|  | **kegg.names** | **all.mapped.symb** | **FoldChange** |
| **MDA-MB-468** | - | 0 | - |
| *AMPA* |  |  |  |
|  |  |  |  |
|  | **kegg.names** | **all.mapped.symb** | **FoldChange** |
| **MDA-MB-468** | 23385 | NCSTN | -1.41337 |
| *Roundup* | 5664 | PSEN2 | 1.17528 |
|  | 3065 | HDAC1 | -1.26378 |
|  | 9541 | CIR1 | 1.73447 |
|  | 1488 | CTBP2 | -1.31652 |
|  | 3280 | HES1 | 1.28645 |
|  | 3516 | RBPJ | -2.82186 |
|  | 2033 | EP300 | -2.0143 |
|  | 6868 | ADAM17 | -1.92828 |
|  |  |  |  |
|  | **kegg.names** | **all.mapped.symb** | **FoldChange** |
| **MCF-7** | - | 0 | - |
| *AMPA* |  |  |  |
|  |  |  |  |
|  | **kegg.names** | **all.mapped.symb** | **FoldChange** |
| **MCF-7** | 9541 | CIR1 | 1.98466 |
| *Roundup* | 3280 | HES1 | 1.96858 |
|  | 22938 | SNW1 | 1.44856 |
|  | 9794 | MAML1 | -1.43547 |
|  | 196403 | DTX3 | 1.31302 |
|  | 4851 | NOTCH1 | 1.4173 |
|  | 18,561,857 | DVL2,DVL3 | 2.70439 |
|  | 28514 | DLL1 | 1.3083 |

Table B: WNT signaling altered by AMPA and Roundup treatments in MDA-MB-468 and MCF-7.

| **04310-Wnt signaling pathway** | | | |
| --- | --- | --- | --- |
|  | **kegg.names** | **all.mapped.symb** | **FoldChange** |
| **MDA-MB-468** | 4316 | MMP7 | -7.78181 |
| *AMPA* | 595 | CCND1 | 2.9637 |
|  | 80319 | CXXC4 | -1.61665 |
|  |  |  |  |
|  | **kegg.names** | **all.mapped.symb** | **FoldChange** |
| **MDA-MB-468** | 55,305,533 | PPP3CA,PPP3CC | -0.30658 |
| *Roundup* | 79,768,323 | FZD3,FZD6 | -3.95466 |
|  | 23002 | DAAM1 | -2.11321 |
|  | 79,768,323 | FZD3,FZD6 | -3.95466 |
|  | 81839 | VANGL1 | -1.62484 |
|  | 595 | CCND1 | 1.87235 |
|  | 4609 | MYC | 2.22488 |
|  | 1488 | CTBP2 | -1.31652 |
|  | 2033 | EP300 | -2.0143 |
|  | 57680 | CHD8 | -1.82124 |
|  | 79718 | TBL1XR1 | -2.03447 |
|  | 1499 | CTNNB1 | -1.32101 |
|  | 5566 | PRKACA | -1.61444 |
|  | 59343 | SENP2 | -1.5124 |
|  | 80319 | CXXC4 | -2.36176 |
|  | 4040 | LRP6 | -1.72543 |
|  |  |  |  |
|  | **kegg.names** | **all.mapped.symb** | **FoldChange** |
| **MCF7** | - | 0 | - |
| *AMPA* |  |  |  |
|  |  |  |  |
|  |  |  |  |
|  | **kegg.names** | **all.mapped.symb** | **FoldChange** |
| **MCF7** | 4088 | SMAD3 | -1.5902 |
| *Roundup* | 55,305,533 | PPP3CA,PPP3CC | -0.73434 |
|  | 817 | CAMK2D | 1.89155 |
|  | 23236 | PLCB1 | -2.42803 |
|  | 18,561,857 | DVL2,DVL3 | 2.70439 |
|  | 79,768,322 | FZD3,FZD4 | -3.73954 |
|  | 81839 | VANGL1 | -1.81805 |
|  | 595 | CCND1 | -2.31627 |
|  | 3725 | JUN | 3.03238 |
|  | 4609 | MYC | -1.57555 |
|  | 8607 | RUVBL1 | -1.73136 |
|  | 8454 | CUL1 | -2.33451 |
|  | 6477 | SIAH1 | 1.31385 |
|  | 59343 | SENP2 | -1.33729 |
|  | 22943 | DKK1 | -2.04525 |
|  | 25805 | BAMBI | -1.88864 |
|  | 27130 | INVS | -1.73232 |

Table C: Hedgehog signaling altered by AMPA and Roundup treatments in MDA-MB-468 and MCF-7.

| **04340-Hedgehog signaling pathway** | | | |
| --- | --- | --- | --- |
|  | **kegg.names** | **all.mapped.symb** | **FoldChange** |
| **MDA-MB-468** | 595 | CCND1 | 2.9637 |
| *AMPA* |  |  |  |
|  |  |  |  |
|  | **kegg.names** | **all.mapped.symb** | **FoldChange** |
| **MDA-MB-468** | 5566 | PRKACA | -1.61444 |
| *Roundup* | 157 | GRK3 | -3.26704 |
|  | 408 | ARRB1 | -1.85475 |
|  | 8405 | SPOP | -1.39725 |
|  | 595 | CCND1 | 1.87235 |
|  |  |  |  |
|  | **kegg.names** | **all.mapped.symb** | **FoldChange** |
| **MCF7** | - | 0 | - |
| *AMPA* |  |  |  |
|  |  |  |  |
|  | **kegg.names** | **all.mapped.symb** | **FoldChange** |
| **MCF7** | 8454 | CUL1 | -2.33451 |
| *Roundup* | 157 | GRK3 | -1.60021 |
|  | 408 | ARRB1 | -4.29066 |
|  | 8405 | SPOP | -1.6621 |
|  | 595 | CCND1 | -2.31627 |

Table D: TGF-Beta signaling altered by AMPA and Roundup treatments in MDA-MB-468 and MCF-7.

| **04350-TGF-beta signaling pathway** | | | |
| --- | --- | --- | --- |
|  | **kegg.names** | **all.mapped.symb** | **FoldChange** |
| **MDA-MB-468** | - | 0 | - |
| *AMPA* |  |  |  |
|  |  |  |  |
|  | **kegg.names** | **all.mapped.symb** | **FoldChange** |
| **MDA-MB-468** | 4052 | LTBP1 | -1.97876 |
| *Roundup* | 93 | ACVR2B | 1.20955 |
|  | 2033 | EP300 | -2.0143 |
|  | 4609 | MYC | 2.22488 |
|  | 7027 | TFDP1 | -1.80913 |
|  | 5519 | PPP2R1B | -2.50382 |
|  | 7048 | TGFBR2 | -1.57831 |
|  | 7046 | TGFBR1 | -1.69915 |
|  | 657 | BMPR1A | -2.27571 |
|  | 653 | BMP5 | -3.65237 |
|  | 4086 | SMAD1 | -1.67638 |
|  |  |  |  |
|  | **kegg.names** | **all.mapped.symb** | **FoldChange** |
| **MCF7** | - | 0 | - |
| *AMPA* |  |  |  |
|  |  |  |  |
|  | **kegg.names** | **all.mapped.symb** | **FoldChange** |
| **MCF7** | 4052 | LTBP1 | -1.65399 |
| *Roundup* | 7057 | THBS1 | -3.83897 |
|  | 8454 | CUL1 | -2.33451 |
|  | 4088 | SMAD3 | -1.5902 |
|  | 6667 | SP1 | 1.34482 |
|  | 4609 | MYC | -1.57555 |
|  | 7027 | TFDP1 | -3.59222 |
|  | 1874 | E2F4 | -1.4231 |
|  | 5933 | RBL1 | -4.344 |
|  | 3398 | ID2 | 2.2762 |
|  | 90 | ACVR1 | 1.4272 |
|  | 36,243,625 | INHBA,INHBB | -3.88588 |
|  | 6198 | RPS6KB1 | -1.72698 |
|  | 5518 | PPP2R1A | -1.35476 |
|  | 5595 | MAPK3 | 1.22416 |
|  | 7046 | TGFBR1 | -1.55386 |
|  | 90,658 | ACVR1,BMPR1B | -0.5689 |
|  | 25805 | BAMBI | -1.88864 |
|  | 705,060,436 | TGIF1,TGIF2 | 0.09653 |

Table E: MAPK signaling altered by AMPA and Roundup treatments in MDA-MB-468 and MCF-7.

| **04010-MAPK signaling pathway** | | | |
| --- | --- | --- | --- |
|  | **kegg.names** | **all.mapped.symb** | **FoldChange** |
| **MDA-MB-468** | 1847 | DUSP5 | 1.66927 |
| *AMPA* | 5606 | MAP2K3 | 1.50176 |
|  | 1647 | GADD45A | 1.54826 |
|  | 355 | FAS | 1.17572 |
|  | 33,033,304 | HSPA1A,HSPA1B | 3.89323 |
|  | 23,162,317 | FLNA,FLNB | -3.93406 |
|  |  |  |  |
|  | **kegg.names** | **all.mapped.symb** | **FoldChange** |
| **MDA-MB-468** | 18,431,844,184,618,400 | DUSP1,DUSP2,DUSP4,DUSP5,DUSP8 | 8.63458 |
| *Roundup* | 8605 | PLA2G4C | 1.27595 |
|  | 5970 | RELA | -1.32536 |
|  | 5604 | MAP2K1 | -1.34598 |
|  | 5566 | PRKACA | -1.61444 |
|  | 4893 | NRAS | -1.85861 |
|  | 2768 | GNA12 | -1.91163 |
|  | 2885 | GRB2 | -1.40373 |
|  | 1956 | EGFR | -1.30483 |
|  | 5154 | PDGFA | 1.32849 |
|  | 9261 | MAPKAPK2 | -1.26407 |
|  | 1649 | DDIT3 | 2.3392 |
|  | 5603 | MAPK13 | -1.25662 |
|  | 5608 | MAP2K6 | -1.9482 |
|  | 5606 | MAP2K3 | 1.45599 |
|  | 1,844,184,618,471,850 | DUSP2,DUSP4,DUSP5,DUSP8 | 7.08969 |
|  | 207 | AKT1 | -1.27526 |
|  | 9175 | MAP3K13 | -1.37583 |
|  | 164,710,912 | GADD45A,GADD45G | 8.60731 |
|  | 7189 | TRAF6 | -1.42077 |
|  | 929 | CD14 | -1.65265 |
|  | 70,467,048 | TGFBR1,TGFBR2 | -3.27746 |
|  | 7132 | TNFRSF1A | -1.33216 |
|  | 6416 | MAP2K4 | -2.70982 |
|  | 408 | ARRB1 | -1.85475 |
|  | 2122 | MECOM | 1.13269 |
|  | 33,033,304 | HSPA1A,HSPA1B | 7.45248 |
|  | 55,305,533 | PPP3CA,PPP3CC | -0.30658 |
|  | 23,162,317 | FLNA,FLNB | -3.47334 |
|  | 8491 | MAP4K3 | -1.67587 |
|  | 2353 | FOS | -2.69185 |
|  | 4609 | MYC | 2.22488 |
|  |  |  |  |
|  | **kegg.names** | **all.mapped.symb** | **FoldChange** |
| **MCF7** | - | 0 | - |
| *AMPA* |  |  |  |
|  |  |  |  |
|  | **kegg.names** | **all.mapped.symb** | **FoldChange** |
| **MCF7** | 184,318,461,847,184 | DUSP1,DUSP4,DUSP5,DUSP6,  DUSP8,DUSP10,DUSP16 | -0.18014 |
| *Roundup* | 8605 | PLA2G4C | 1.41421 |
|  | 47,915,970 | NFKB2,RELA | 0.08107 |
|  | 5595 | MAPK3 | 1.22416 |
|  | 8649 | LAMTOR3 | 1.22905 |
|  | 6237 | RRAS | 2.05348 |
|  | 10125 | RASGRP1 | -1.86564 |
|  | 6654 | SOS1 | -2.9132 |
|  | 2768 | GNA12 | -1.39387 |
|  | 776 | CACNA1D | -1.14924 |
|  | 5154 | PDGFA | 1.47476 |
|  | 2258 | FGF13 | 1.20082 |
|  | 994 | CDC25B | 1.6969 |
|  | 1649 | DDIT3 | 11.22 |
|  | 2005 | ELK4 | -2.62144 |
|  | 5536 | PPP5C | -1.20695 |
|  | 1,846,184,718,501,120 | DUSP4,DUSP5,DUSP8,  DUSP10,DUSP16 | -1.05683 |
|  | 5494 | PPM1A | 1.21228 |
|  | 207 | AKT1 | -1.45136 |
|  | 9344 | TAOK2 | -1.46177 |
|  | 1,647,461,610,912 | GADD45A,GADD45B,GADD45G | 7.60341 |
|  | 7046 | TGFBR1 | -1.55386 |
|  | 355 | FAS | 1.44028 |
|  | 5058 | PAK1 | 1.91433 |
|  | 4215 | MAP3K3 | -1.36407 |
|  | 6416 | MAP2K4 | -2.18441 |
|  | 408 | ARRB1 | -4.29066 |
|  | 3,303,330,433,103,310 | HSPA1A,HSPA1B,HSPA6,HSPA8 | 5.7113 |
|  | 3727 | JUND | 2.44368 |
|  | 3725 | JUN | 3.03238 |
|  | 55,305,533 | PPP3CA,PPP3CC | -0.73434 |
|  | 23162 | MAPK8IP3 | 1.36088 |
|  | 4609 | MYC | -1.57555 |
|  | 468 | ATF4 | 1.33087 |
|  | 2872 | MKNK2 | 1.68252 |

Table F: Jak-STAT signaling altered by AMPA and Roundup treatments in MDA-MB-468 and MCF-7.

| **04630-Jak-STAT signaling pathway** | | | |
| --- | --- | --- | --- |
|  | **kegg.names** | **all.mapped.symb** | **FoldChange** |
| **MDA-MB-468** | 9655 | SOCS5 | -1.68149 |
| *AMPA* | 3,953,116,379 | LEPR,IL22RA2 | -1.49221 |
|  | 4170 | MCL1 | 1.54614 |
|  | 595 | CCND1 | 2.9637 |
|  |  |  |  |
|  | **kegg.names** | **all.mapped.symb** | **FoldChange** |
| **MDA-MB-468** | 4609 | MYC | 2.22488 |
| *Roundup* | 207 | AKT1 | -1.27526 |
|  | 8503 | PIK3R3 | -1.39341 |
|  | 2885 | GRB2 | -1.40373 |
|  | 88,359,655 | SOCS2,SOCS5 | -0.73062 |
|  | 2033 | EP300 | -2.0143 |
|  | 6774 | STAT3 | -1.52988 |
|  | 9063 | PIAS2 | -2.48894 |
|  | 34,543,455,357,035,900 | IFNAR1,IFNAR2,IL6R,IL13RA2,LEPR  ,LIFR,PRLR,OSMR,IL22RA2 | -9.10308 |
|  | 4170 | MCL1 | 1.9665 |
|  | 5292 | PIM1 | 2.64244 |
|  | 595 | CCND1 | 1.87235 |
|  | 5781 | PTPN11 | -1.73574 |
|  | 345,236,003,976 | IFNA21,IL15,LIF | 4.08332 |
|  |  |  |  |
|  | **kegg.names** | **all.mapped.symb** | **FoldChange** |
| **MCF7** | - | 0 | - |
| *AMPA* |  |  |  |
|  |  |  |  |
|  | **kegg.names** | **all.mapped.symb** | **FoldChange** |
| **MCF7** | 4609 | MYC | -1.57555 |
| *Roundup* | 207 | AKT1 | -1.45136 |
|  | 52,958,503 | PIK3R1,PIK3R3 | -3.51345 |
|  | 6654 | SOS1 | -2.9132 |
|  | 122809 | SOCS4 | -1.77555 |
|  | 345,434,593,566,357 | IFNAR1,IFNGR1,IL4R,IL6R,PRLR,OSMR,IL23R | -3.51488 |
|  | 5292 | PIM1 | 2.25608 |
|  | 595 | CCND1 | -2.31627 |
|  | 1026 | CDKN1A | 1.54068 |
|  | 2475 | MTOR | -1.36336 |
|  | 344,134,523,976 | IFNA4,IFNA21,LIF | 3.87752 |

Table G: PI3K-Akt signaling altered by AMPA and Roundup treatments in MDA-MB-468 and MCF-7.

| **04151-PI3K-Akt signaling pathway** | | | |
| --- | --- | --- | --- |
|  | **kegg.names** | **all.mapped.symb** | **FoldChange** |
| **MDA-MB-468** | 3791 | KDR | -6.94965 |
| *AMPA* | 2997 | GYS1 | -1.44154 |
|  | 595 | CCND1 | 2.9637 |
|  | 10971 | YWHAQ | -1.28612 |
|  | 2,847,422 | ANGPT1,VEGFA | -2.89139 |
|  | 2335 | FN1 | -3.98049 |
|  | 64764 | CREB3L2 | 1.43167 |
|  | 4170 | MCL1 | 1.54614 |
|  | 5,959,134 | CCND1,CCNE2 | 1.05963 |
|  |  |  |  |
|  | **kegg.names** | **all.mapped.symb** | **FoldChange** |
| **MDA-MB-468** | 57521 | RPTOR | -1.21876 |
| *Roundup* | 207 | AKT1 | -1.27526 |
|  | 5170 | PDPK1 | -1.25453 |
|  | 19,563,791 | EGFR,KDR | -11.20536 |
|  | 23566 | LPAR3 | 1.7081 |
|  | 4609 | MYC | 2.22488 |
|  | 4193 | MDM2 | -2.66374 |
|  | 2997 | GYS1 | -2.18839 |
|  | 595 | CCND1 | 1.87235 |
|  | 5970 | RELA | -1.32536 |
|  | 752,910,971 | YWHAB,YWHAQ | -3.16721 |
|  | 284,194,230,825,154 | ANGPT1,EFNA1,HGF,PDGFA | 2.85496 |
|  | 2885 | GRB2 | -1.40373 |
|  | 4893 | NRAS | -1.85861 |
|  | 5519 | PPP2R1B | -2.50382 |
|  | 5563 | PRKAA2 | -1.80201 |
|  | 200186 | CRTC2 | 1.58138 |
|  | 8503 | PIK3R3 | -1.39341 |
|  | 5604 | MAP2K1 | -1.34598 |
|  | 6850 | SYK | -1.26938 |
|  | 59345 | GNB4 | -1.62336 |
|  | 3,673,367,536,783,680 | ITGA2,ITGA3,ITGA5,ITGAV | -4.74384 |
|  | 36,883,694 | ITGB1,ITGB6 | -3.64339 |
|  | 2335 | FN1 | -3.69669 |
|  | 34,543,455,357,056,100 | IFNAR1,IFNAR2,IL6R,PRLR,OSMR | -6.68513 |
|  | 3452 | IFNA21 | 1.20515 |
|  | 672 | BRCA1 | -2.21952 |
|  | 4170 | MCL1 | 1.9665 |
|  | 5586 | PKN2 | -2.10423 |
|  | 6446 | SGK1 | -1.70915 |
|  | 5,959,134 | CCND1,CCNE2 | -1.02277 |
|  |  |  |  |
|  | **kegg.names** | **all.mapped.symb** | **FoldChange** |
| **MCF7** | - | 0 | - |
| *AMPA* |  |  |  |
|  |  |  |  |
|  | **kegg.names** | **all.mapped.symb** | **FoldChange** |
| **MCF7** | 6198 | RPS6KB1 | -1.72698 |
| *Roundup* | 57521 | RPTOR | -1.35047 |
|  | 2475 | MTOR | -1.36336 |
|  | 7248 | TSC1 | 1.53369 |
|  | 207 | AKT1 | -1.45136 |
|  | 5170 | PDPK1 | -1.27301 |
|  | 52,958,503 | PIK3R1,PIK3R3 | -3.51345 |
|  | 3667 | IRS1 | -2.49024 |
|  | 3815 | KIT | 1.17529 |
|  | 4609 | MYC | -1.57555 |
|  | 2309 | FOXO3 | 1.39091 |
|  | 2997 | GYS1 | -2.22936 |
|  | 595 | CCND1 | -2.31627 |
|  | 92579 | G6PC3 | -1.49653 |
|  | 5970 | RELA | -1.37797 |
|  | 752,910,971 | YWHAB,YWHAQ | -2.9106 |
|  | 1,942,225,851,547,420 | EFNA1,FGF13,PDGFA,VEGFA | 6.64949 |
|  | 6654 | SOS1 | -2.9132 |
|  | 117145 | THEM4 | -1.36324 |
|  | 5518 | PPP2R1A | -1.35476 |
|  | 3320 | HSP90AA1 | -1.34752 |
|  | 54541 | DDIT4 | 1.53507 |
|  | 200186 | CRTC2 | 1.57985 |
|  | 1026 | CDKN1A | 1.54068 |
|  | 1027 | CDKN1B | 1.33111 |
|  | 10,171,019 | CDK2,CDK4 | -3.52415 |
|  | 5595 | MAPK3 | 1.22416 |
|  | 36,733,678 | ITGA2,ITGA5 | -4.638 |
|  | 36,933,694 | ITGB5,ITGB6 | -4.01412 |
|  | 705,770,597,148 | THBS1,THBS3,TNXB | -1.07954 |
|  | 34,543,566,357,056,100 | IFNAR1,IL4R,IL6R,PRLR,OSMR | -6.10927 |
|  | 34,413,452 | IFNA4,IFNA21 | 2.4541 |
|  | 672 | BRCA1 | -5.67086 |
|  | 4,681,385,958,664,760 | ATF4,CREB1,CREB5,CREB3L2 | -1.79553 |
|  | 4602 | MYB | -2.98368 |
|  | 23678 | SGK3 | -1.9303 |
|  | 5,958,989,134 | CCND1,CCNE1,CCNE2 | -10.22877 |

Table H: Ras signaling altered by AMPA and Roundup treatments in MDA-MB-468 and MCF-7.

| **04014-Ras signaling pathway** | | | |
| --- | --- | --- | --- |
|  | **kegg.names** | **all.mapped.symb** | **FoldChange** |
| **MDA-MB-468** | 6464 | SHC1 | 1.23454 |
| *AMPA* | 2,847,422 | ANGPT1,VEGFA | -2.89139 |
|  | 3791 | KDR | -6.94965 |
|  | 5336 | PLCG2 | 2.41017 |
|  |  |  |  |
|  | **kegg.names** | **all.mapped.symb** | **FoldChange** |
| **MDA-MB-468** | 5604 | MAP2K1 | -1.34598 |
| *Roundup* | 4893 | NRAS | -1.85861 |
|  | 2885 | GRB2 | -1.40373 |
|  | 207 | AKT1 | -1.27526 |
|  | 284,194,230,825,154 | ANGPT1,EFNA1,HGF,PDGFA | 2.85496 |
|  | 19,563,791 | EGFR,KDR | -11.20536 |
|  | 9846 | GAB2 | 2.52185 |
|  | 5781 | PTPN11 | -1.73574 |
|  | 5898 | RALA | 1.3771 |
|  | 5970 | RELA | -1.32536 |
|  | 8605 | PLA2G4C | 1.27595 |
|  | 5878 | RAB5C | -1.19987 |
|  | 883,194,621,015,622 | SYNGAP1,RASAL2,RASA4,RASA3 | 2.26049 |
|  | 8503 | PIK3R3 | -1.39341 |
|  | 59345 | GNB4 | -1.62336 |
|  | 8315 | BRAP | 1.48726 |
|  | 5566 | PRKACA | -1.61444 |
|  |  |  |  |
|  | **kegg.names** | **all.mapped.symb** | **FoldChange** |
| **MCF7** | - | 0 | - |
| *AMPA* |  |  |  |
|  |  |  |  |
|  | **kegg.names** | **all.mapped.symb** | **FoldChange** |
| **MCF7** | 5595 | MAPK3 | 1.22416 |
| *Roundup* | 6237 | RRAS | 2.05348 |
|  | 207 | AKT1 | -1.45136 |
|  | 399694 | SHC4 | -2.16206 |
|  | 1,942,225,851,547,420 | EFNA1,FGF13,PDGFA,VEGFA | 6.64949 |
|  | 3815 | KIT | 1.17529 |
|  | 9846 | GAB2 | 2.01497 |
|  | 7074 | TIAM1 | -1.53036 |
|  | 58,985,899 | RALA,RALB | 2.787 |
|  | 5337 | PLD1 | 1.73837 |
|  | 5970 | RELA | -1.37797 |
|  | 8605 | PLA2G4C | 1.41421 |
|  | 2114 | ETS2 | 1.6582 |
|  | 5869 | RAB5B | 1.44379 |
|  | 10125 | RASGRP1 | -1.86564 |
|  | 1,015,622,821,100,270 | RASA4,RASA3,RASA4B | 4.0107 |
|  | 52,958,503 | PIK3R1,PIK3R3 | -3.51345 |
|  | 382 | ARF6 | 1.21823 |
|  | 6654 | SOS1 | -2.9132 |
|  | 805,808 | CALM2,CALM3 | -2.88519 |
|  | 5058 | PAK1 | 1.91433 |
|  | 29110 | TBK1 | -1.80757 |

Table I: Cell cycle altered by AMPA and Roundup treatments in MDA-MB-468 and MCF-7.

| **04110-Cell cycle** | | | |
| --- | --- | --- | --- |
|  | **kegg.names** | **all.mapped.symb** | **FoldChange** |
| **MDA-MB-468** | 25847 | ANAPC13 | 1.23834 |
| *AMPA* | 10971 | YWHAQ | -1.28612 |
|  | 1647 | GADD45A | 1.54826 |
|  |  |  |  |
|  | **kegg.names** | **all.mapped.symb** | **FoldChange** |
| **MDA-MB-468** | 41,724,175 | MCM3,MCM6 | -3.98753 |
| *Roundup* | 4175 | MCM6 | -1.82966 |
|  | 4172 | MCM3 | -2.15787 |
|  | 5111 | PCNA | -2.59038 |
|  | 752,910,971 | YWHAB,YWHAQ | -3.16721 |
|  | 4193 | MDM2 | -2.66374 |
|  | 2033 | EP300 | -2.0143 |
|  | 9700 | ESPL1 | -1.47626 |
|  | 824,327,127 | SMC1A,SMC1B | -0.59717 |
|  | 6502 | SKP2 | -2.72852 |
|  | 164,710,912 | GADD45A,GADD45G | 8.60731 |
|  | 8317 | CDC7 | -1.99808 |
|  | 1022 | CDK7 | 1.41379 |
|  | 9134 | CCNE2 | -2.89512 |
|  | 595 | CCND1 | 1.87235 |
|  | 4609 | MYC | 2.22488 |
|  | 1871 | E2F3 | -1.55193 |
|  | 3065 | HDAC1 | -1.26378 |
|  | 7027 | TFDP1 | -1.80913 |
|  |  |  |  |
|  | **kegg.names** | **all.mapped.symb** | **FoldChange** |
| **MCF7** | - | 0 | - |
| *AMPA* |  |  |  |
|  |  |  |  |
|  | **kegg.names** | **all.mapped.symb** | **FoldChange** |
| **MCF7** | 417,141,724,173,417 | MCM2,MCM3,MCM4,MCM5,MCM6,MCM7 | -18.34083 |
| *Roundup* | 4998 | ORC1 | -1.93171 |
|  | 258,475,143,364,682 | ANAPC13,ANAPC5,ANAPC1 | -2.45386 |
|  | 8454 | CUL1 | -2.33451 |
|  | 4176 | MCM7 | -2.43615 |
|  | 4175 | MCM6 | -3.60524 |
|  | 4174 | MCM5 | -2.39331 |
|  | 4173 | MCM4 | -3.17951 |
|  | 4172 | MCM3 | -4.67229 |
|  | 4171 | MCM2 | -2.05433 |
|  | 5111 | PCNA | -5.47179 |
|  | 752,910,971 | YWHAB,YWHAQ | -2.9106 |
|  | 1111 | CHEK1 | -1.7673 |
|  | 1026 | CDKN1A | 1.54068 |
|  | 5591 | PRKDC | -2.97235 |
|  | 6502 | SKP2 | -3.32078 |
|  | 9700 | ESPL1 | -1.73692 |
|  | 8243 | SMC1A | -2.09591 |
|  | 1,647,461,610,912 | GADD45A,GADD45B,GADD45G | 7.60341 |
|  | 1027 | CDKN1B | 1.33111 |
|  | 4088 | SMAD3 | -1.5902 |
|  | 8317 | CDC7 | -2.55017 |
|  | 994 | CDC25B | 1.6969 |
|  | 8318 | CDC45 | -2.54137 |
|  | 990 | CDC6 | -3.23631 |
|  | 993 | CDC25A | -2.40053 |
|  | 890 | CCNA2 | -2.16991 |
|  | 1022 | CDK7 | 1.37178 |
|  | 1017 | CDK2 | -2.04264 |
|  | 1019 | CDK4 | -1.48151 |
|  | 8,989,134 | CCNE1,CCNE2 | -7.9125 |
|  | 595 | CCND1 | -2.31627 |
|  | 5933 | RBL1 | -4.344 |
|  | 1874 | E2F4 | -1.4231 |
|  | 4609 | MYC | -1.57555 |
|  | 7027 | TFDP1 | -3.59222 |
|  | 18,691,871 | E2F1,E2F3 | -3.02979 |
|  | 5933 | RBL1 | -4.344 |

Table J: Apostosis altered by AMPA and Roundup treatments in MDA-MB-468 and MCF-7.

| **04210-Apoptosis** | | | |
| --- | --- | --- | --- |
|  | **kegg.names** | **all.mapped.symb** | **FoldChange** |
| **MDA-MB-468** | 8795 | TNFRSF10B | 1.43596 |
| *AMPA* | 355 | FAS | 1.17572 |
|  | 5366 | PMAIP1 | 1.47177 |
|  | 1647 | GADD45A | 1.54826 |
|  | 355 | FAS | 1.17572 |
|  | 15,151,519 | CTSV,CTSO | 0.72999 |
|  | 3710 | ITPR3 | -1.16208 |
|  | 7846 | TUBA1A | -1.46443 |
|  | 4170 | MCL1 | 1.54614 |
|  |  |  |  |
|  | **kegg.names** | **all.mapped.symb** | **FoldChange** |
| **MDA-MB-468** | 9131 | AIFM1 | -1.407 |
| *Roundup* | 329 | BIRC2 | -1.98009 |
|  | 5970 | RELA | -1.32536 |
|  | 317 | APAF1 | -2.47035 |
|  | 823 | CAPN1 | -1.47334 |
|  | 207 | AKT1 | -1.27526 |
|  | 8503 | PIK3R3 | -1.39341 |
|  | 7132 | TNFRSF1A | -1.33216 |
|  | 8795 | TNFRSF10B | 1.39465 |
|  | 8743 | TNFSF10 | -1.58924 |
|  | 4001 | LMNB1 | -2.59316 |
|  | 142 | PARP1 | -2.38293 |
|  | 2081 | ERN1 | 1.64533 |
|  | 164,710,912 | GADD45A,GADD45G | 8.60731 |
|  | 2353 | FOS | -2.69185 |
|  | 107,515,091,514 | CTSC,CTSD,CTSL | -1.22752 |
|  | 1649 | DDIT3 | 2.3392 |
|  | 37,083,710 | ITPR1,ITPR3 | 0.09737 |
|  | 5170 | PDPK1 | -1.25453 |
|  | 60 | ACTB | -1.52606 |
|  | 78,461,037,684,790 | TUBA1A,TUBA1B,TUBA1C | -4.79514 |
|  | 4170 | MCL1 | 1.9665 |
|  | 4893 | NRAS | -1.85861 |
|  | 5604 | MAP2K1 | -1.34598 |
|  |  |  |  |
|  | **kegg.names** | **all.mapped.symb** | **FoldChange** |
| **MCF7** | - | 0 | - |
| *AMPA* |  |  |  |
|  |  |  |  |
|  | **kegg.names** | **all.mapped.symb** | **FoldChange** |
| **MCF7** | 1676 | DFFA | -1.58508 |
| *Roundup* | 5970 | RELA | -1.37797 |
|  | 207 | AKT1 | -1.45136 |
|  | 52,958,503 | PIK3R1,PIK3R3 | -3.51345 |
|  | 8737 | RIPK1 | -1.9694 |
|  | 87,948,795 | TNFRSF10C,TNFRSF10B | 3.19115 |
|  | 355 | FAS | 1.44028 |
|  | 4001 | LMNB1 | -2.49632 |
|  | 142 | PARP1 | -2.17357 |
|  | 2081 | ERN1 | 3.3709 |
|  | 1,647,461,610,912 | GADD45A,GADD45B,GADD45G | 7.60341 |
|  | 581 | BAX | 1.41385 |
|  | 3725 | JUN | 3.03238 |
|  | 5366 | PMAIP1 | 1.51348 |
|  | 1509 | CTSD | -1.42286 |
|  | 1649 | DDIT3 | 11.22 |
|  | 468 | ATF4 | 1.33087 |
|  | 3710 | ITPR3 | -1.12694 |
|  | 5170 | PDPK1 | -1.27301 |
|  | 637 | BID | -1.32387 |
|  | 835 | CASP2 | -1.48384 |
|  | 78,461,037,684,790 | TUBA1A,TUBA1B,TUBA1C | -5.07174 |
|  | 5595 | MAPK3 | 1.22416 |
|  |  |  |  |

Table K: Base excision repair system altered by AMPA and Roundup treatments in MDA-MB-468 and MCF-7.

| **03410-Base excision repair** | | | |
| --- | --- | --- | --- |
|  | **kegg.names** | **all.mapped.symb** | **FoldChange** |
| **MDA-MB-468** | - | 0 | - |
| *AMPA* |  |  |  |
|  |  |  |  |
|  | **kegg.names** | **all.mapped.symb** | **FoldChange** |
| **MDA-MB-468** | 27301 | APEX2 | -1.60136 |
| *Roundup* | 2237 | FEN1 | -1.94905 |
|  | 142 | PARP1 | -2.38293 |
|  | 3146 | HMGB1 | -2.06694 |
|  | 5111 | PCNA | -2.59038 |
|  | 2237 | FEN1 | -1.94905 |
|  |  |  |  |
|  | **kegg.names** | **all.mapped.symb** | **FoldChange** |
| **MCF7** | - | 0 | - |
| *AMPA* |  |  |  |
|  |  |  |  |
|  | **kegg.names** | **all.mapped.symb** | **FoldChange** |
| **MCF7** | 27301 | APEX2 | -1.46954 |
| *Roundup* | 2237 | FEN1 | -2.62275 |
|  | 142 | PARP1 | -2.17357 |
|  | 3980 | LIG3 | 1.43578 |
|  | 54,265,427 | POLE,POLE2 | -4.96738 |
|  | 10714 | POLD3 | -2.95037 |
|  | 7374 | UNG | -2.50307 |
|  | 3146 | HMGB1 | -2.79473 |
|  | 5111 | PCNA | -5.47179 |
|  | 27343 | POLL | 1.39738 |
|  | 4968 | OGG1 | -1.27013 |
|  | 8930 | MBD4 | -1.65589 |
|  | 55247 | NEIL3 | -1.88933 |
|  |  |  |  |

Table L: Nucleotide excision repair system altered by AMPA and Roundup treatments in MDA-MB-468 and MCF-7.

| **03420-Nucleotide excision repair** | | | |
| --- | --- | --- | --- |
|  | **kegg.names** | **all.mapped.symb** | **FoldChange** |
| **MDA-MB-468** | - | 0 | - |
| *AMPA* |  |  |  |
|  |  |  |  |
|  | **kegg.names** | **all.mapped.symb** | **FoldChange** |
| **MDA-MB-468** | 2967 | GTF2H3 | -1.87898 |
| *Roundup* | 1022 | CDK7 | 1.41379 |
|  | 8451 | CUL4A | -1.36692 |
|  | 5111 | PCNA | -2.59038 |
|  | 6117 | RPA1 | -1.86887 |
|  |  |  |  |
|  | **kegg.names** | **all.mapped.symb** | **FoldChange** |
| **MCF7** | - | 0 | - |
| *AMPA* |  |  |  |
|  |  |  |  |
|  | **kegg.names** | **all.mapped.symb** | **FoldChange** |
| **MCF7** | 2967 | GTF2H3 | -1.95178 |
| *Roundup* | 728340 | GTF2H2C | -2.01246 |
|  | 5,982,598,359,845,980 | RFC2,RFC3,RFC4,RFC5 | -8.15281 |
|  | 5111 | PCNA | -5.47179 |
|  | 54,265,427 | POLE,POLE2 | -4.96738 |
|  | 2073 | ERCC5 | 1.40596 |
|  | 10714 | POLD3 | -2.95037 |
|  | 6118 | RPA2 | -1.3303 |
|  | 8451 | CUL4A | -1.34642 |
|  | 1161 | ERCC8 | -1.90062 |
|  | 1022 | CDK7 | 1.37178 |

**Table M: Mismatch repair system altered by AMPA and Roundup treatments in MDA-MB-468 and MCF-**7.

| **03430-Mismatch repair** | | | |
| --- | --- | --- | --- |
|  | **kegg.names** | **all.mapped.symb** | **FoldChange** |
| **MDA-MB-468** | - | 0 | 0 |
| *AMPA* |  |  |  |
|  |  |  |  |
|  | **kegg.names** | **all.mapped.symb** | **FoldChange** |
| **MDA-MB-468** | 5111 | PCNA | -2.59038 |
| *Roundup* | 6117 | RPA1 | -1.86887 |
|  |  |  |  |
|  | **kegg.names** | **all.mapped.symb** | **FoldChange** |
| **MCF7** | - | 0 | - |
| *AMPA* |  |  |  |
|  |  |  |  |
|  | **kegg.names** | **all.mapped.symb** | **FoldChange** |
| **MCF7** | 4436 | MSH2 | -2.35992 |
| *Roundup* | 27030 | MLH3 | -1.63873 |
|  | 5111 | PCNA | -5.47179 |
|  | 10714 | POLD3 | -2.95037 |
|  | 6118 | RPA2 | -1.3303 |
|  | 9156 | EXO1 | -4.00237 |
|  | 5,982,598,359,845,980 | RFC2,RFC3,RFC4,RFC5 | -8.15281 |


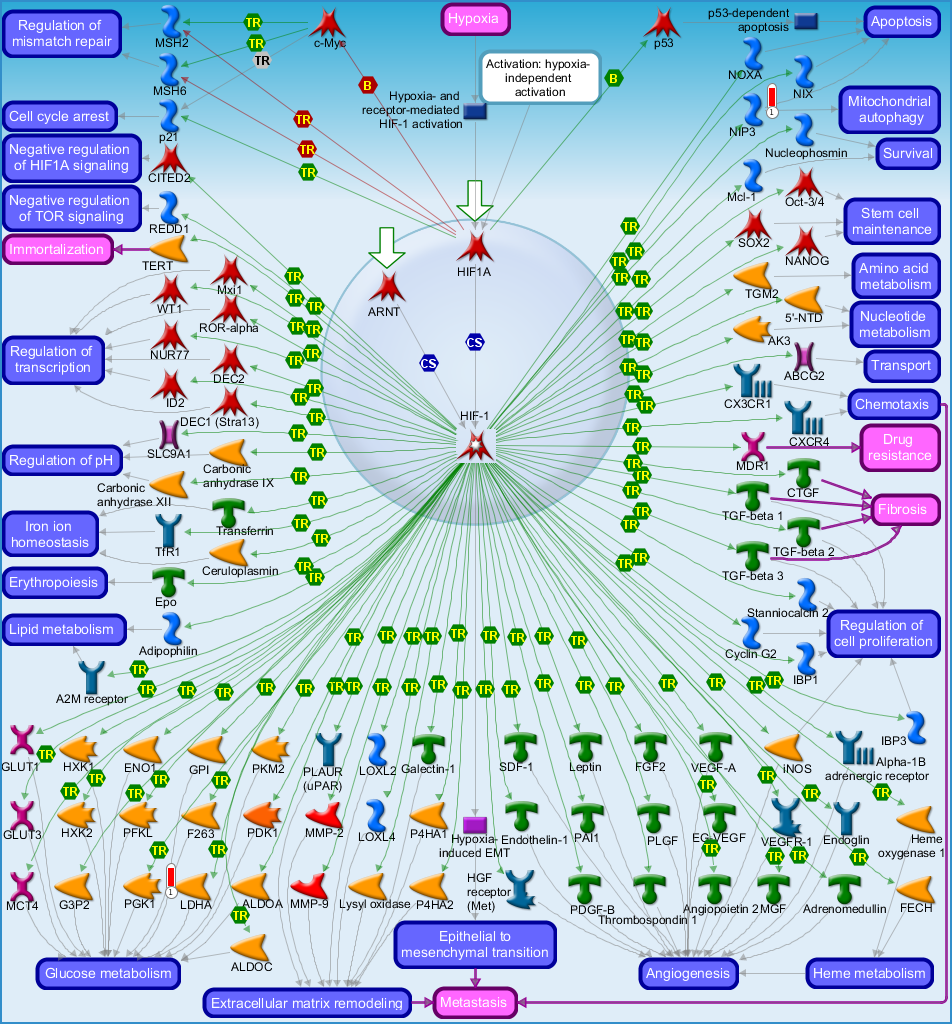


***Figure A:*** *Transcription- HIF 1 targets pathways from common genes expressed in both cell lines and treatments.* Figure generate by *MetaCore* from *Clarivate Analytic*s.


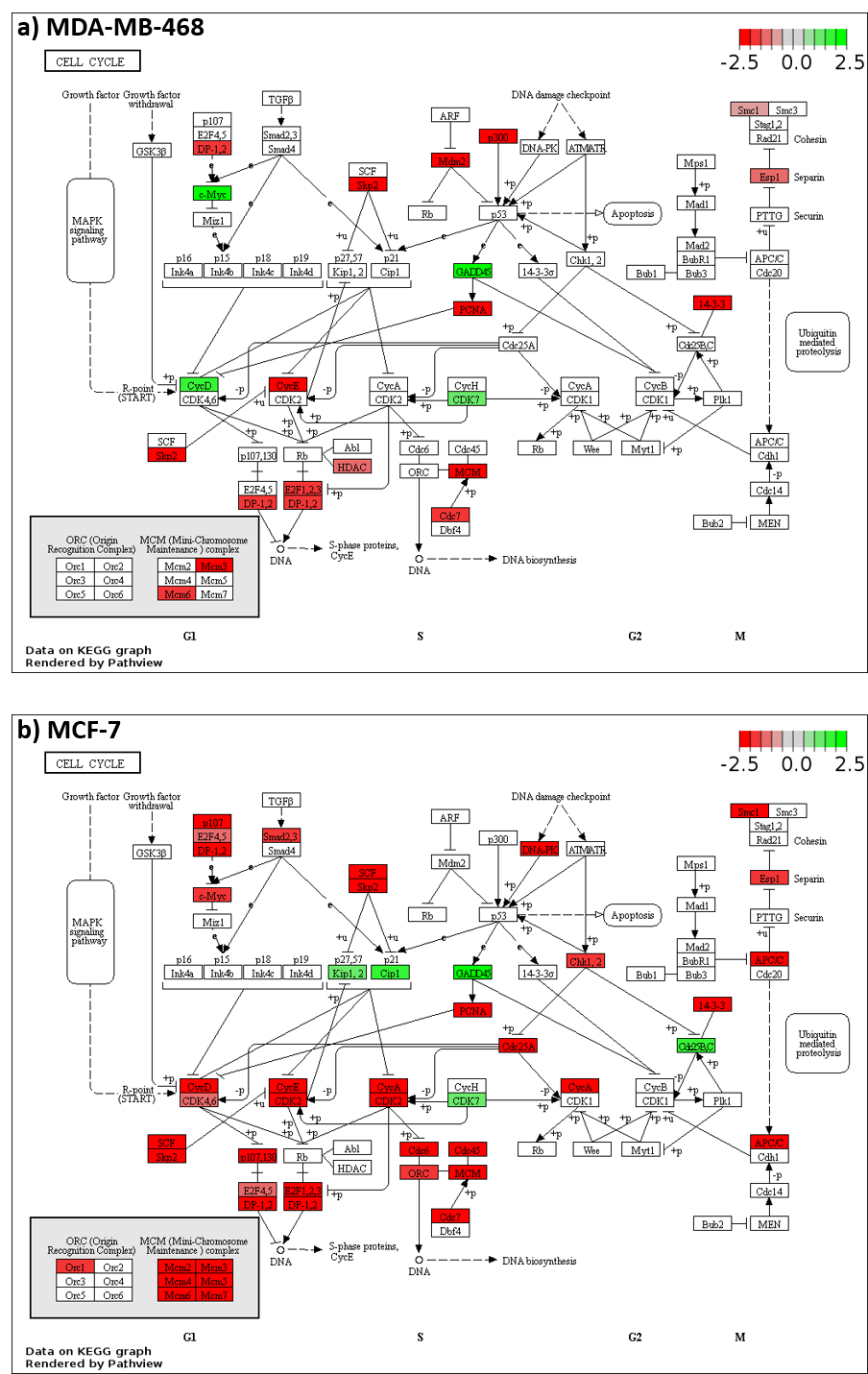


**Figure B:** Gene expression alterations in the cell cycle pathway on MDA-MB-468 (ER-) (a) and MCF-7 (ER+) (b) cell lines, after treatment with Roundup®. Figure generated by KEGG database resource.


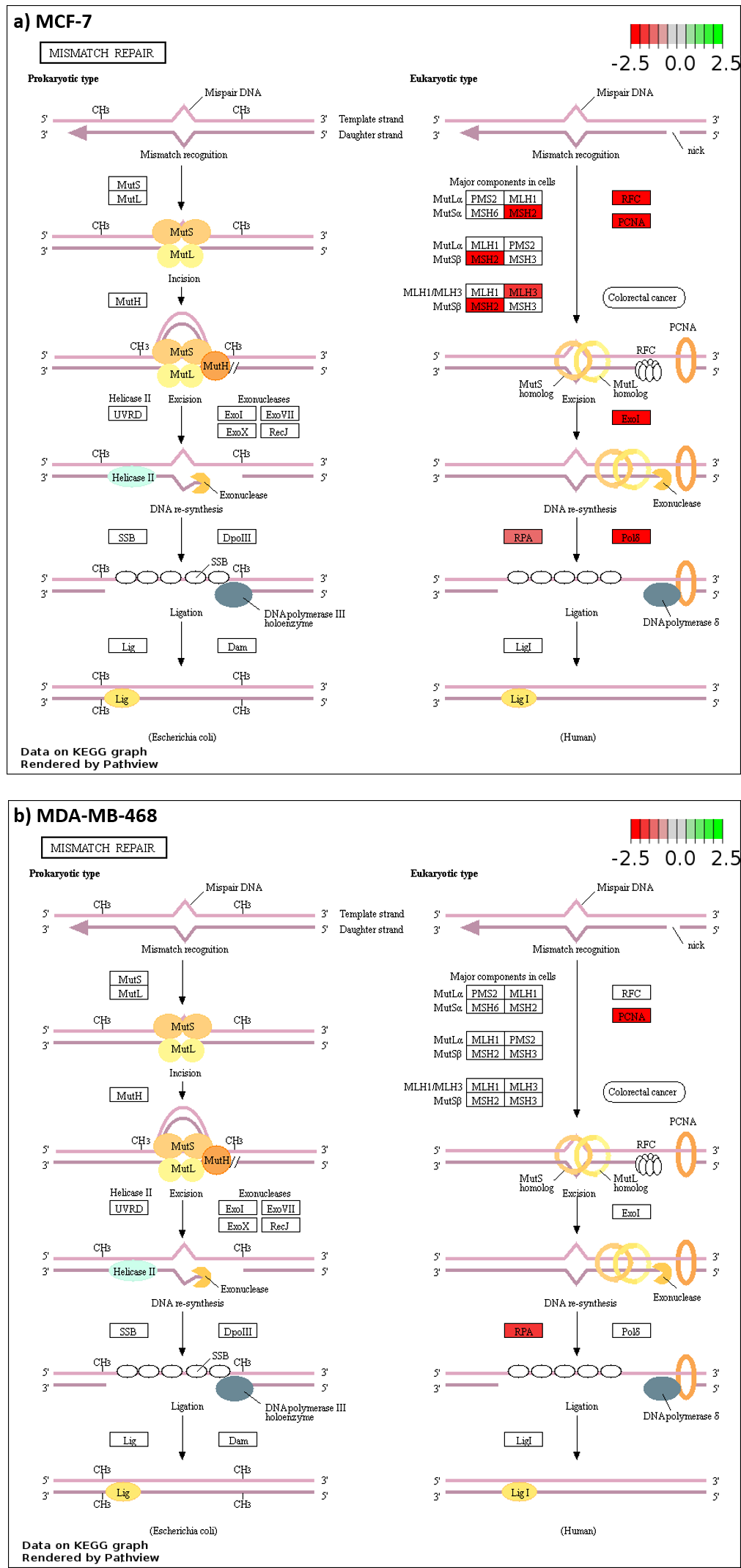


**Figure C:** Mismatch repair altered by Roundup treatment in a) MCF-7 and b) MDA-MB-468. Figure generated by KEGG database resource.


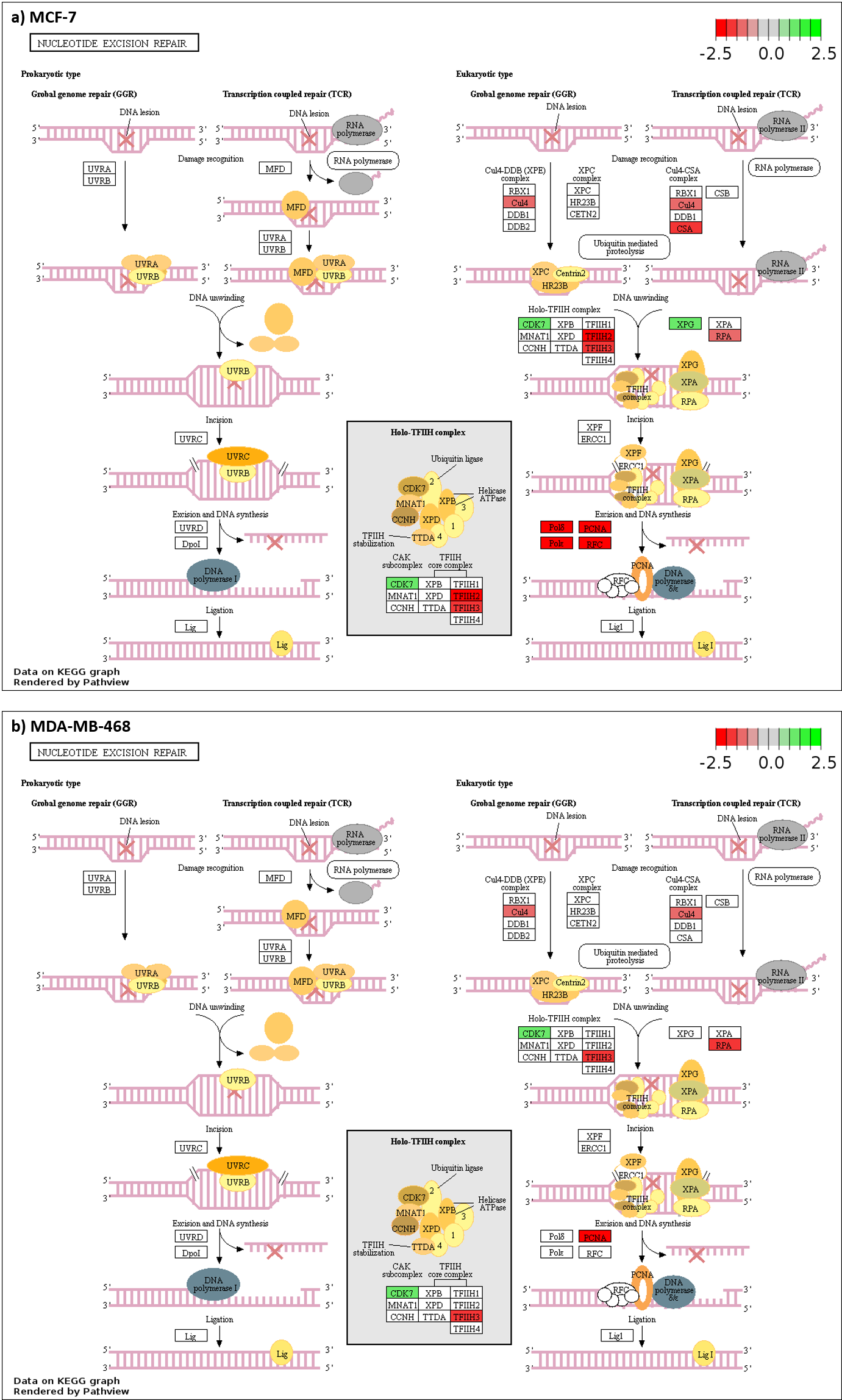


**Figure D:** Nucleotide excision repair altered by Roundup treatment in a) MCF-7 and b) MDA-MB-468. Figure generated by KEGG database resource.


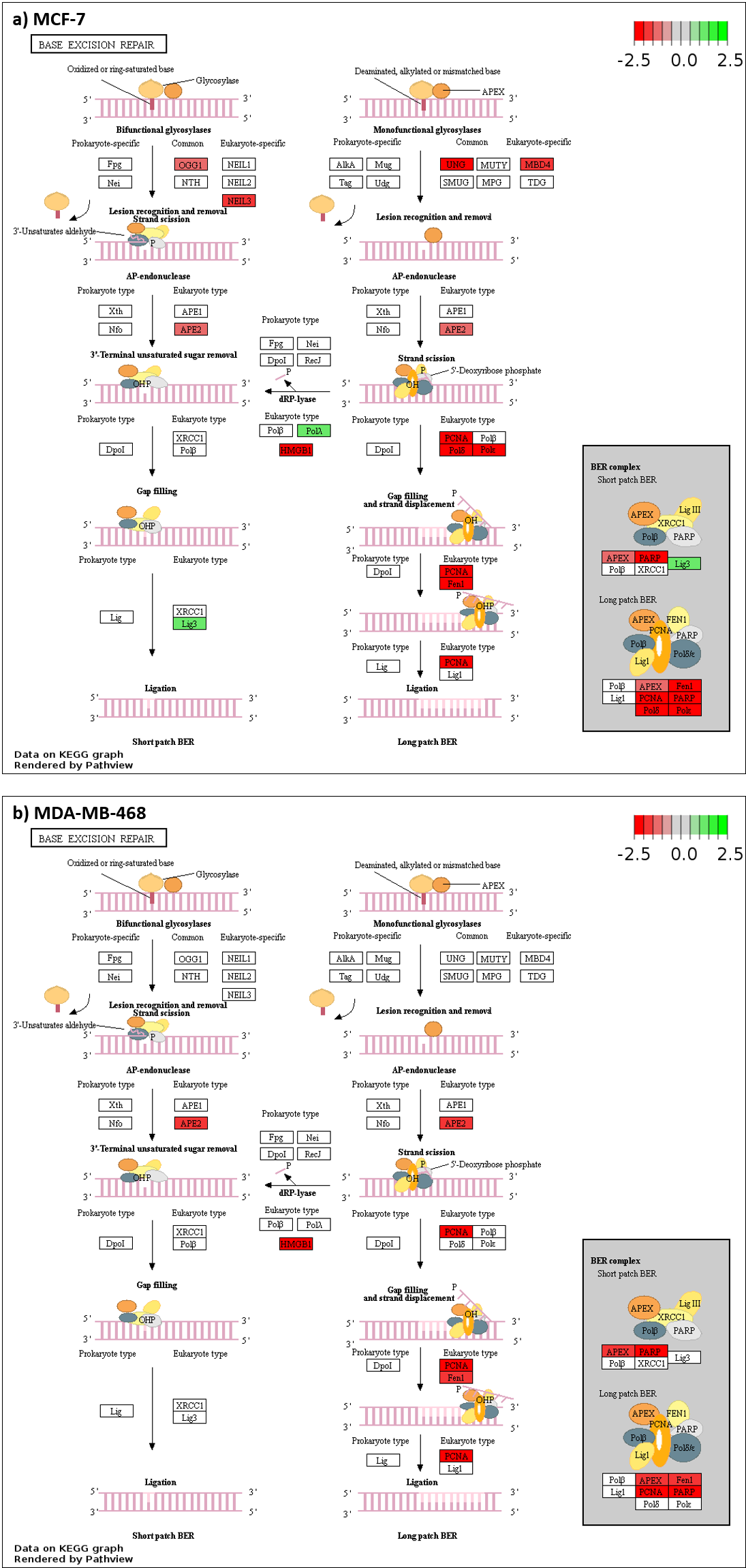


**Figure E:** Base excision repair altered by Roundup treatment in a) MCF-7 and b) MDA-MB-468. Figure generated by KEGG database resource.
